# Midterm Follow-Up of Haemodynamic Performance of the St. Jude Medical Trifecta Aortic Bioprosthesis in Young Patients Under 65

**DOI:** 10.1186/1749-8090-10-S1-A307

**Published:** 2015-12-16

**Authors:** JA Chacko, J Edlin, AH Sepehripour, SG Ambekar, KS Lall

**Affiliations:** 1 St.Bartholomew's Hospital, London, UK

## Background/Introduction

The St. Jude Medical Trifecta aortic supra-annular bioprosthesis is regarded as the next generation in pericardial stented tissue valves. The unique design of tissue leaflets attached to the exterior of the valve stent provides unrivalled in-vivo mean gradients and haemodynamics.

## Aims/Objectives

The aim of this prospective study was to evaluate midterm haemodynamic performance of valve implanted into patients under 65.

## Method

Thirty consecutive patients undergoing aortic valve replacement using the St. Jude Medical Trifecta valve at a single UK centre over a 48-month period were included in this study. Patients undergoing concomitant cardiac procedures were included. All implanted valves were 21, 23, 25 and 27 mm in size. Assessment of haemodynamic function was carried out using transthoracic echocardiography pre-operatively and at follow-up, as well as transoesophageal echocardiography intra-operatively.

## Results

The study population consisted of 30 patients (22 male, 8 female). Mean age was 52.63 ± 11.8 years. Implanted valve sizes were 21 mm (n = 9), 23 mm (n = 11), 25 mm (n = 7) and 27 mm (n = 3). Overall mean post-operative pressure gradients were 11.3 ± 5.8 mmHg (mean) and 19.54 ± 9.5 mmHg (peak). Subgroup mean post-operative pressure gradients were 18.37 ± 4.6 mmHg, 9.8 ± 2.2 mmHg, 9.46 ± 2.54 mmHg, 7.78 ± 2.3 mmHg for the 21, 23, 25 and 27 mm cohort respectively. Overall mean post-operative left ventricular ejection fraction was 54.9 ± 3.3%. Overall mean effective orifice area was 1.95 ± 0.7 cm2.

## Discussion/Conclusion

These results of our experience demonstrate excellent haemodynamic performance of the Trifecta bioprosthetic valve in young patients under 65.

